# Antimicrobial Peptides: An Emerging Category of Therapeutic Agents

**DOI:** 10.3389/fcimb.2016.00194

**Published:** 2016-12-27

**Authors:** Margit Mahlapuu, Joakim Håkansson, Lovisa Ringstad, Camilla Björn

**Affiliations:** ^1^Promore Pharma AB, Karolinska Institutet Science ParkSolna, Sweden; ^2^The Lundberg Laboratory for Diabetes Research, Department of Molecular and Clinical Medicine, The Sahlgrenska Academy at University of GothenburgGothenburg, Sweden; ^3^SP Technical Research Institute of Sweden, Chemistry, Materials, and SurfacesBorås, Sweden

**Keywords:** AMP, antimicrobial peptide, anti-infectives, antibiotic resistance, therapeutic agents

## Abstract

Antimicrobial peptides (AMPs), also known as host defense peptides, are short and generally positively charged peptides found in a wide variety of life forms from microorganisms to humans. Most AMPs have the ability to kill microbial pathogens directly, whereas others act indirectly by modulating the host defense systems. Against a background of rapidly increasing resistance development to conventional antibiotics all over the world, efforts to bring AMPs into clinical use are accelerating. Several AMPs are currently being evaluated in clinical trials as novel anti-infectives, but also as new pharmacological agents to modulate the immune response, promote wound healing, and prevent post-surgical adhesions. In this review, we provide an overview of the biological role, classification, and mode of action of AMPs, discuss the opportunities and challenges to develop these peptides for clinical applications, and review the innovative formulation strategies for application of AMPs.

## Introduction

**Figure 1:**
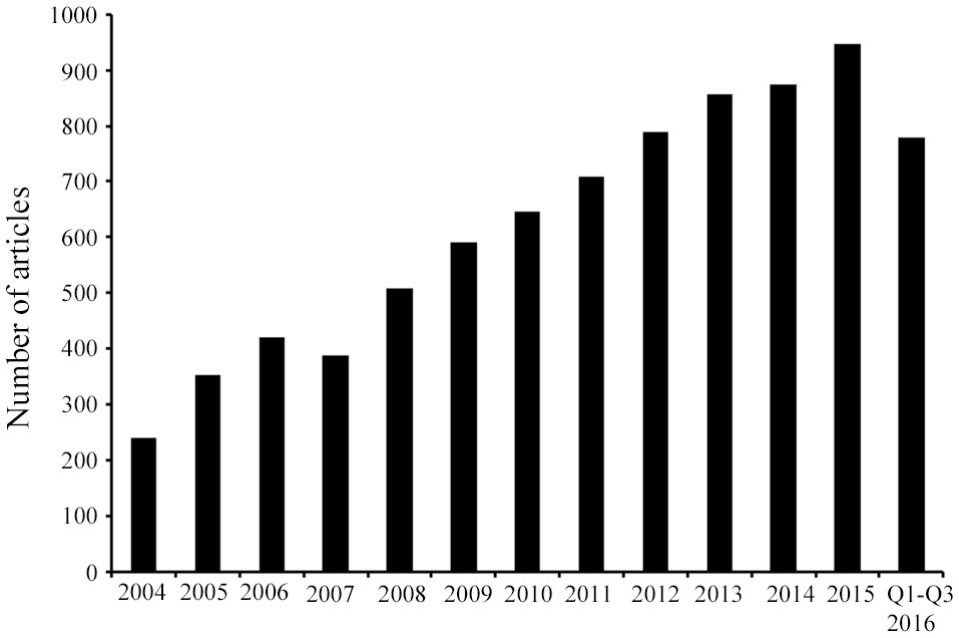
**Published research on AMPs identified from 2004 until September 2016**. Article counts were carried out after searching in PubMed using the following key words: antimicrobial peptides, AMPs, and/or host defense peptides. The search results demonstrate that in the last decade the AMP research field has progressively expanded as represented by the continuous increase in the number of articles. Q, quarter.

The rapidly increasing resistance toward conventional antibiotics suggests that, without urgent action, we are heading for a “post-antibiotic era,” in which the previously effective therapeutic strategies are no longer relevant. Due to the limited number of available antibiotics, and the similarities in their activity spectrum as well as mode of action, intensive nonclinical and clinical research is now invested into identification of new and non-conventional anti-infective therapies, including adjunctive or preventive approaches such as antibodies targeting a virulence factor, probiotics, and vaccines (Czaplewski et al., 2016). Interestingly, the antimicrobial peptides (AMPs) have rapidly captured attention as novel drug candidates (Figure 1). AMPs have been found virtually in all organisms and they display remarkable structural and functional diversity. Besides direct antimicrobial activity, AMPs carry immunomodulatory properties (Fjell et al., 2012), which make them especially interesting compounds for the development of novel therapeutics. There are encouraging examples of AMPs already introduced into the market, and many AMPs are currently being tested in clinical trials (Fox, 2013), which provide a reason for optimism for introduction of novel AMP-based drugs in several indication areas.

With no attempt to provide a comprehensive overview with regards to all types of AMPs identified from different sources, this review focuses on applied therapeutic aspects with the emphasis of AMPs being evaluated as potential pharmacological agents.

## Biological role and classification of AMPs

AMPs are evolutionary conserved in the genome and produced by all life forms, from prokaryotes to humans (Hancock, 2000). In higher organisms, AMPs constitute important components of the innate immunity, protecting the host against infections. In contrast, bacteria produce AMPs in order to kill other bacteria competing for the same ecological niche (Hassan et al., 2012). Many AMPs exhibit an extraordinarily broad range of antimicrobial activity covering both Gram-positive and Gram-negative bacteria as well as fungi, viruses, and unicellular protozoa (Hancock and Diamond, 2000; Reddy et al., 2004; Marr et al., 2006). Besides having a direct antimicrobial activity, several AMPs display ability to modulate the innate immune responses of the host and thereby indirectly promote pathogen clearance (Hancock and Sahl, 2006; Yeung et al., 2011). The widespread distribution and abundance of AMPs in all multicellular organisms underscores their critical role in innate immunity (Zasloff, 2002; Hancock et al., 2012). Their importance is further demonstrated by the increased infection susceptibility of mice genetically modified to lack the gene encoding for the mouse analog of the human AMP LL-37 (Nizet et al., 2001) and of humans with diseases associated with reduced AMP production such as atopic dermatitis (Ong et al., 2002).

AMPs in nature are produced either by ribosomal translation of mRNA or by nonribosomal peptide synthesis (Hancock and Chapple, 1999). While nonribosomally synthesized peptides are mainly produced by bacteria, the ribosomally synthesized AMPs are genetically encoded and produced by all species of life, bacteria included (Hancock and Chapple, 1999). Compared to peptides of nonribosomal origin that have been known for several decades and whereof many are used as antibiotics (e.g., polymyxins and gramicidin S), the ribosomally synthesized AMPs have more recently been recognized for their critical role in innate immunity and for their therapeutic potential (Hancock and Chapple, 1999; Hancock, 2000).

In mammals, AMPs are found primarily within granules of neutrophils and in secretions from epithelial cells covering skin and mucosal surfaces (Boman, 1995; Hancock and Chapple, 1999). In many cases, AMPs are encoded in clusters in the genome and co-expressed, resulting in multiple AMPs accumulating at a single site (Lai and Gallo, 2009). Notably, many AMPs are produced as inactive precursors requiring proteolytic cleavage to become active (Bals, 2000). Their regulation is therefore not only dependent on their own expression but also on the abundance of appropriate proteases (Lai and Gallo, 2009). In multicellular organisms, some AMPs are constitutively expressed, stored at high concentrations as inactive precursors in granules and released locally at infection and inflammation sites, whereas the expression of others is induced in response to pathogen-associated molecular patterns (PAMPs) or cytokines (Hancock and Diamond, 2000; Lai and Gallo, 2009).

**Figure 2:**
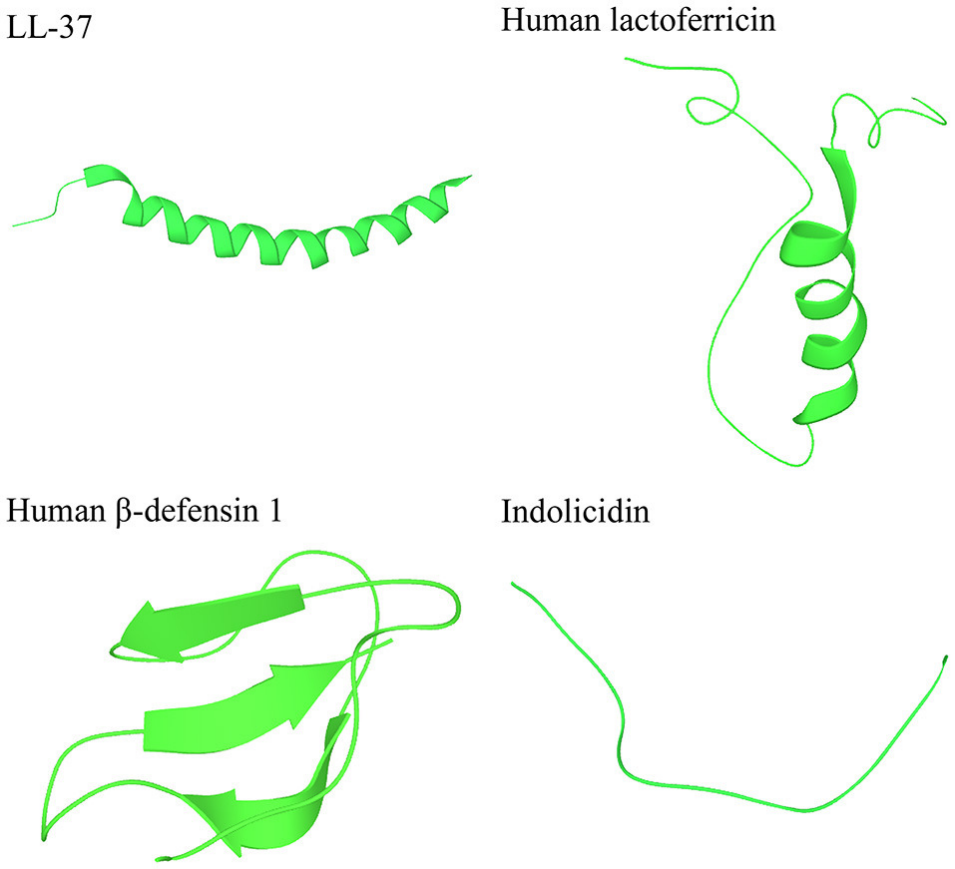
**Peptides representing the three main categories of the secondary structures of AMPs**. LL-37 and human lactoferricin represent α-helical peptides, human β-defensin 1 represents β-sheet peptides, and indolocidin represents extended/random-coil structures. Structures are from Protein Data Bank in Europe (PDB id codes 2k6o, 1z6v, 1kj5, and 1g89).

Several databases exist for natural AMPs, today covering more than 2000 peptides (Wang, 2015). Most AMPs are relatively short, commonly consisting of 10–50 amino acids, display an overall positive charge ranging from +2 to +11, and contain a substantial proportion (typically 50%) of hydrophobic residues (Yeaman and Yount, 2003; Hancock and Sahl, 2006; Pasupuleti et al., 2012). AMPs are commonly classified based on their secondary structure into α-helical, β-sheet, or peptides with extended/random-coil structure (Takahashi et al., 2010; Nguyen et al., 2011; Pasupuleti et al., 2012), with most AMPs belonging to the first two categories (Figure 2). α-helical peptides are often unstructured in aqueous solution, but adopt an amphipathic helical structure in contact with a biological membrane (Yeaman and Yount, 2003; Pasupuleti et al., 2012). Two of the most studied peptides in this group are: (i) LL-37 (Epand and Vogel, 1999; Pasupuleti et al., 2012), which is produced as an inactive precursor in the 18-kDa human cathelicidin antimicrobial protein (hCAP18), present in neutrophils and epithelial cells (Lai and Gallo, 2009), and (ii) human lactoferricin, which is derived by proteolytic cleavage of the antimicrobial and immunomodulatory iron-binding glycoprotein lactoferrin, present in milk and exocrine secretions (Hunter et al., 2005; Legrand et al., 2005). β-sheet peptides are stabilized by disulphide bonds (Powers and Hancock, 2003; Yount et al., 2006) and are organized to create an amphipathic molecule (Yeaman and Yount, 2003). Due to their rigid structure, the β-sheet peptides are more ordered in aqueous solution and do not undergo as drastic conformational change as helical peptides upon membrane interaction (Yeaman and Yount, 2003). The best-studied β-sheet peptides are the defensins—a large group of AMPs, which are produced as inactive precursors in neutrophils, macrophages, and epithelial cells (Lai and Gallo, 2009; Pasupuleti et al., 2012). A small portion of the natural AMPs belong to the third class of extended/random-coil peptides which lack secondary structure and often contain a high content of arginine, proline, tryptophan, and/or histidine residues (Takahashi et al., 2010; Nguyen et al., 2011). Similarly to other AMPs, many of the extended peptides fold into amphipathic structures after contact with a membrane (Nguyen et al., 2011). One of the best studied peptides in this group is indolicidin, produced by bovine leukocytes (Powers and Hancock, 2003).

## Mechanism of action of AMPs

### Interaction with bacterial membrane

Many AMPs display a direct and rapid antimicrobial activity by causing disruption of the physical integrity of the microbial membrane and/or by translocating across the membrane into the cytoplasm of bacteria to act on intracellular targets (Hancock and Sahl, 2006). It is widely accepted that membrane interaction is a key factor for the direct antimicrobial activity of AMPs, both when the membrane itself is targeted and when an intracellular target must be reached by means of translocation (Jenssen et al., 2006; Nguyen et al., 2011; Yeung et al., 2011; Malmsten, 2016). Electrostatic forces between the cationic AMPs and the negatively charged bacterial surface are critical determinants for this interaction between peptides and microbial membrane (Yeaman and Yount, 2003; Giuliani et al., 2007; Yeung et al., 2011; Ebenhan et al., 2014). Bacteria are commonly divided into two families, Gram-positive and Gram-negative, based on the differences in cell envelope structure. In Gram-positive bacteria, the cytoplasmic membrane is surrounded by a thick peptidoglycan layer, whereas the cytoplasmic membrane of Gram-negative bacteria is surrounded by a thin peptidoglycan layer as well as an outer membrane (Lin and Weibel, 2016). The cytoplasmic membranes of both Gram-positive and Gram-negative bacteria are rich in the phospholipids phosphatidylglycerol, cardiolipin, and phosphatidylserine, which have negatively charged head groups, highly attractive for positively charged AMPs (Yeaman and Yount, 2003; Ebenhan et al., 2014). The presence of teichoic acids in the cell wall of Gram-positive bacteria and lipopolysaccharides (LPS) in the outer membrane of Gram-negative bacteria provide additional electronegative charge to the bacterial surface (Lai and Gallo, 2009; Ebenhan et al., 2014).

The fundamental differences between microbial and mammalian membranes protect mammalian cells against AMPs and enable selective action of these peptides (Yeaman and Yount, 2003). In contrast to bacteria, the cytoplasmic membrane of mammalian cells is rich in the zwitterionic phospholipids phosphatidylethanolamine, phosphatidylcholine, and sphingomyelin, providing a membrane with a neutral net charge (Yeaman and Yount, 2003; Ebenhan et al., 2014). There is also an asymmetric distribution of phospholipids in mammalian membranes, with the zwitterionic phospholipids being present in the outer leaflet, while phospholipids with negatively charged head groups, if present, are localized in the inner leaflet facing the cytoplasm (Zasloff, 2002; Yeaman and Yount, 2003; Lai and Gallo, 2009). Therefore, interactions between AMPs and mammalian cell membrane occur mainly via hydrophobic interactions, which are relatively weak compared to the electrostatic interactions taking place between AMPs and bacterial membranes. Furthermore, mammalian cell membranes, unlike those of microbes, have a high content of cholesterol (Yeaman and Yount, 2003; Lai and Gallo, 2009). The cholesterol is proposed to reduce the activity of AMPs via stabilization of the phospholipid bilayer (Zasloff, 2002). Notably, bacterial cells typically have an inside-negative transmembrane potential between −130 and −150 mV in contrast to mammalian cells, where the potential ranges from −90 to −110 mV (Yeaman and Yount, 2003; Matsuzaki, 2009; Ebenhan et al., 2014). A stronger negative membrane potential in bacteria may also contribute to selectivity of AMPs between bacterial vs. mammalian cells (Yeaman and Yount, 2003).

### Membrane disruption and intracellular targets in bacterial cells

In order to reach the cytoplasmic membrane of Gram-negative bacteria, AMPs have to first translocate through the outer membrane. This outer membrane constitutes a permeability barrier for many macromolecules, partly due to the divalent cations Ca^2+^ and Mg^2+^ that bind to the phosphate groups of the inner core of LPS and thereby provide stabilization of the outer leaflet (Clifton et al., 2015). AMPs are proposed to be translocated through this outer membrane via so called self-promoted uptake (Hancock, 1997; Hancock and Chapple, 1999; Giuliani et al., 2007). This model suggests that, due to greater affinity for the LPS, AMPs displace the divalent cations and bind to the LPS. By being bulky, the AMPs then cause transient cracks and permeabilize the outer membrane, thereby permitting passage of the peptide itself across the membrane.

**Figure 3:**
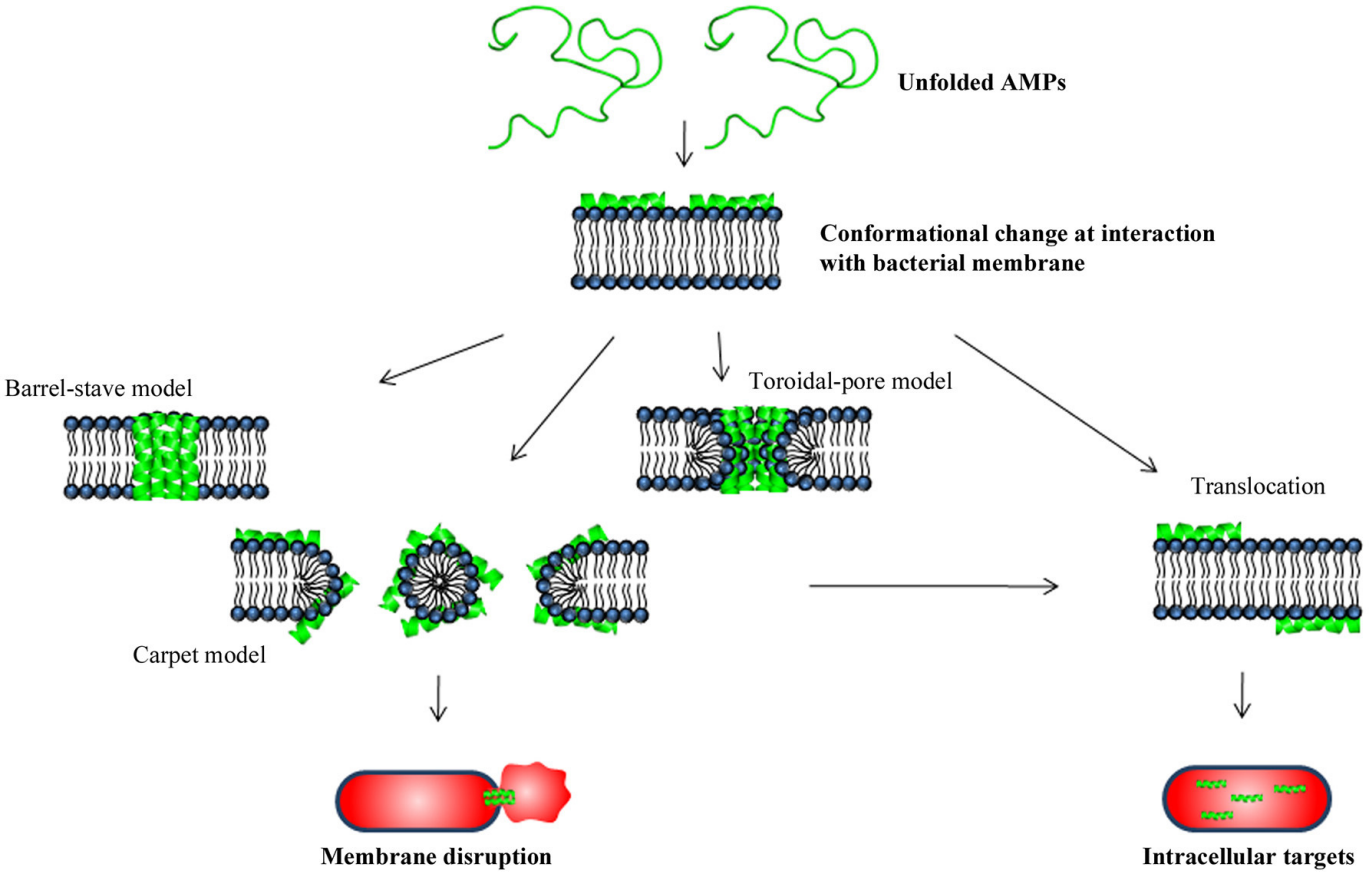
**Schematic illustration of bacterial killing mechanisms by AMPs**.

In contact with the cytoplasmic membrane, the AMPs form an amphipathic secondary structure (if not already present) essential for interaction with the cell membrane (Ebenhan et al., 2014). The charged domains of the peptide allow for interaction with the hydrophilic head groups of the phospholipids, while the hydrophobic domains of the peptide interact with the hydrophobic core of the lipid bilayer, thereby driving the AMP deeper into the membrane (Ebenhan et al., 2014). Several models have been proposed describing the next events occurring at the bacterial cytoplasmic membrane, which ultimately lead to membrane permeabilization (Figure 3; Brogden, 2005; Toke, 2005; Nguyen et al., 2011). According to the “barrel-stave model,” the peptides insert perpendicularly into the bilayer while recruitment of additional peptides subsequently results in formation of a peptide-lined transmembrane pore. In this pore, the peptides are aligned with the hydrophobic side facing the lipid core of the membrane and the hydrophilic regions facing the interior region of the pore. According to the “toroidal-pore model,” insertion of peptides forces the phospholipid to bend continuously from one leaflet to the other, resulting in a pore lined by both peptides and the head groups of the phospholipids. Finally, in the “carpet model,” accumulation of peptides on the membrane surface causes tension in the bilayer that ultimately leads to disruption of the membrane and formation of micelles.

Membrane permeabilization by AMPs is suggested to initially lead to leakage of ions and metabolites, depolarization of the transmembrane potential with subsequent membrane dysfunction (e.g., impaired osmotic regulation and inhibition of respiration), and ultimately, membrane rupture and rapid lysis of microbial cells (Yeaman and Yount, 2003; Brogden, 2005; Eckert, 2011).

Besides leading to membrane dysfunction and disruption, membrane permeabilization is important for translocation of certain AMPs into the cytoplasm, where they target key cellular processes including DNA/RNA and protein synthesis, protein folding, enzymatic activity, and/or cell wall synthesis (Figure 3; Yeaman and Yount, 2003; Brogden, 2005; Yount et al., 2006; Nguyen et al., 2011).

Notably, it is suggested that bacterial death caused by AMPs could be a result of multiple and complementary actions, referred to as multi-hit mechanism. This strategy helps to increase the efficiency of AMPs and to evade resistance development (Zhang et al., 2000; Yeaman and Yount, 2003; Peschel and Sahl, 2006; Nguyen et al., 2011). It is likely that the mode of action of individual AMPs varies depending on parameters such as peptide concentration, target bacterial species, as well as tissue localization and growth phase of the bacteria (Yeaman and Yount, 2003; Jenssen et al., 2006). Importantly, regardless of the exact mode of action and target site, the antibacterial activity of AMPs is dependent on the interaction with microbial membrane (Jenssen et al., 2006; Yeung et al., 2011).

Interestingly, the membrane-destabilizing activity of AMPs is also utilized in so called Artilysins, which have recently shown potential to effectively target resistant and persistent Gram-negative infections. Artilysins are engineered fusions of bacteriophage-encoded endolysins, which degrade peptidoglycans of the bacterial cells wall, with specific AMPs, which facilitate the transduction of the endolysin through the protective outer membrane of Gram-negative pathogens to reach its substrate (Briers et al., 2014a,b; Briers and Lavigne, 2015; Defraine et al., 2016).

### Immunomodulatory activities

**Figure 4:**
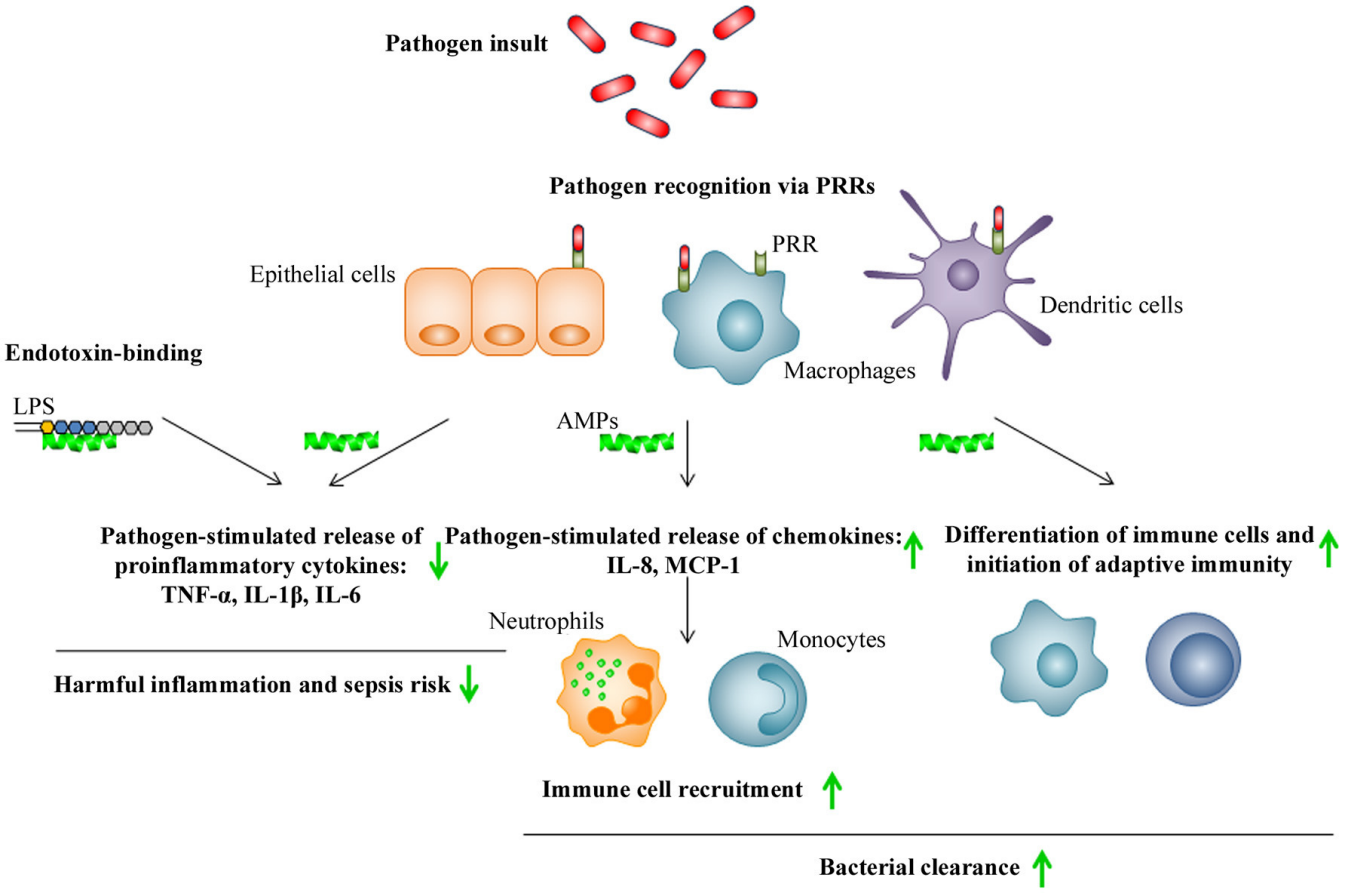
**Schematic illustration of immunomodulatory activities of AMPs**. Pathogen recognition via pathogen recognition receptors (PRRs), such as TLRs, by epithelial cells, macrophages, and dendritic cells, leads to killing via phagocytosis as well as release of proinflammatory cytokines and chemokines by these cells, that subsequently stimulates the recruitment of additional immune cells to the site of infection. In addition, pathogen insult will lead to maturation of dendritic cells and subsequent initiation of adaptive immunity. AMPs indirectly promote pathogen clearance by stimulating chemotaxis and immune cell differentiation, while also preventing harmful inflammation and sepsis by inhibition of proinflammatory cytokine release and direct scavenging of bacterial endotoxins such as LPS. Up- or down-regulation of responses by AMPs is indicated by green arrows.

Recently published analysis of the available patent information referring to the therapeutic use of AMPs covering the period from 2003 to 2015 concluded that most of the claimed AMPs were characterized not only as potent antibiotics, but also as effective modulators of inflammation or neutralizers of pathogenic toxins (Kosikowska and Lesner, 2016). The broad range of immunomodulatory activities exerted by AMPs include stimulation of chemotaxis, modulation of immune cell differentiation and initiation of adaptive immunity, together contributing to the bacterial clearance of the host (Figure 4). The immunomodulatory activities further include suppression of toll-like receptors (TLR)- and/or cytokine-mediated production of proinflammatory cytokines and anti-endotoxin activity, together preventing excessive and harmful proinflammatory responses including sepsis (Håversen et al., 2002; Davidson et al., 2004; Mookherjee et al., 2006; Lai and Gallo, 2009; van der Does et al., 2010; Yeung et al., 2011; Figure 4). As an example, LL-37 and bovine lactoferricin have been reported to inhibit the LPS (TLR4)-induced secretion of TNF-α and IL-6, respectively, in THP-1 cells and, in addition, LL-37 suppresses the LTA (TLR2)- and LPS (TLR4)-induced production of TNF-α, IL-1β, IL-6, and IL-8 in primary monocytes (Mattsby-Baltzer et al., 1996; Mookherjee et al., 2006). Several mechanisms have been proposed to explain these immunomodulatory actions of AMPs on mammalian cells (Lai and Gallo, 2009). In the “alternate ligand model,” the AMPs bind directly to specific cell surface receptors thereby inducing receptor signaling. In the “membrane disruption model,” the AMPs locally modify the part of membrane that contains the receptor and thereby indirectly alter the activation state and function of the receptor. In the “trans-activation model,” the AMPs cause release of a membrane-bound factor, which could then bind to its receptor (Lai and Gallo, 2009). In addition, scavenging of the endotoxin LPS by AMPs has been suggested, preventing LPS from binding the TLR4 and triggering inflammation (Lai and Gallo, 2009).

## Potential for development of bacterial resistance to AMPs

The widespread bacterial resistance development toward AMPs has generally been considered to be unlikely due to the AMPs' mechanism of action involving attacking multiple low-affinity targets rather than one defined, high-affinity target characteristic for conventional antibiotics, which makes it more difficult for target microbes to defend themselves by a single resistance mechanism (Peschel and Sahl, 2006; Lai and Gallo, 2009; Fjell et al., 2012). In particular, given that the bacterial cell membrane is the primary target of AMPs, it is challenging for microbes to preserve the cell membrane functional and structural integrity while at the same time avoiding the membrane-disrupting activity of AMPs (Lai and Gallo, 2009). However, considerable experimental data has recently emerged describing mechanisms by which bacteria may develop resistance toward AMPs under selection pressure *in vitro* (Pränting et al., 2008; Lofton et al., 2013), warranting further investigations of the potential risks of bacterial AMP resistance. The detailed discussion on the underlying mechanisms and consequences of microbial resistance to AMPs is beyond the scope of this review, and the reader is referred to the excellent recent review by Andersson et al. (2016).

## AMPs as therapeutic agents

The rapid bactericidal activity of AMPs makes them promising candidates for therapeutic anti-infectives. Furthermore, several AMPs have a broad range of action, which is an advantage in certain therapeutic areas, such as complicated skin and soft tissue infections, where a rapidly increasing incidence of polymicrobial infections involving both Gram-positive and Gram-negative organisms has been reported over the last decade (Dryden, 2010). To date, only a few AMPs are approved for clinical use, with polymyxins, introduced already in the 1950s, being the most well characterized (Falagas and Kasiakou, 2005; Zavascki et al., 2007; Landman et al., 2008). Polymyxins are last-resort drugs for intravenous treatment of drug-resistant infections caused by Gram-negative pathogens, but they are also applied as topical formulations in the prevention and treatment of local infections (Zavascki et al., 2007).

There are numerous AMPs currently under clinical development for the treatment against various bacterial pathogens (Table 1) with pexiganan and omiganan, derived from animal immune components, and synthetic LTX-109, being the most well described. Pexiganan, a 22-amino-acid membrane disruptor analog of the *Xenopus* peptide magainin, has been evaluated as a topical cream for treating bacterial infections associated with diabetic foot ulcers in two phase III clinical trials (Clinical trial identifiers: NCT00563394, NCT00563433) (Lamb and Wiseman, 1998; Lipsky et al., 2008), and additional clinical trials are currently ongoing. Omiganan is a derivative of indolicidin, which was isolated from bovine neutrophils, and this AMP has been assessed as a topical gel in clinical trials for catheter infections (NCT00231153) and rosacea (NCT01784133). LTX-109 is a synthetic antimicrobial peptidomimetic, which has been to date evaluated for local application in uncomplicated Gram-positive skin infections (NCT01223222), impetigo (NCT01803035), and in subjects nasally colonized with *S. aureus* (NCT01158235) (Nilsson et al., 2015). While most of the AMPs, including the above mentioned pexiganan, omiganan, and LTX-109, are developed for local application, there are a few AMPs aimed for systemic administration. hLF1-11 is a cationic fragment comprising N-terminal amino acids 1-11 of human lactoferricin, and this peptide is developed for the intravenous treatment of life-threatening bacterial and fungal infections in immunocompromised stem cell transplant recipients (NCT00509938) (Velden et al., 2009). In addition to hLF1-11, there are several other AMPs being developed for treatment of fungal infections. For example, novexatin, a cyclic and highly cationic peptide based on human α- and β-defensins, is targeting stubborn fungal infections in toenails (Fox, 2013) while CZEN-002, a dimeric peptide sequentially derived from α-melanocyte-stimulating hormone (α-MSH), is targeting vaginal candidiasis (Fjell et al., 2012).

**Table 1:** **Selected AMPs in clinical phase of development**.

**AMP**	**Description**	**Phase**	**Indication**	**Administration**	**Clinical trial identifier if available**
Pexiganan (MSI-78)	Analog of magainin (skin of African clawed frog)	Phase III	Infected diabetic foot ulcers	Topical cream	NCT00563394, NCT00563433
Omiganan	Derived from indolicidin (bovine)	Phase II/III	Catheter infections and rosacea	Topical gel	NCT00231153, NCT01784133
Lytixar (LTX-109)	Synthetic antimicrobial peptidomimetic	Phase I/II	Uncomplicated Gram-positive skin infections, impetigo, and nasal colonization with *S. aureus*	Topical hydrogel	NCT01223222, NCT01803035, NCT01158235
hLF1-11	Derived from lactoferricin (human)	Phase I/II	Bacteraemia and fungal infections in immunocompromized haematopoetic stem cell transplant recipients	Intravenous treatment (in saline)	NCT00509938
Novexatin (NP-213)	Derived from defensins (human)	Phase II	Onychomycosis (fungal nail infection)	Topical brush-on-treatment	
CZEN-002	Dimeric octamer derived from α-MSH (human)	Phase IIb	Vaginal candidiasis	Vaginal gel	
LL-37	LL-37 (human)	Phase I/II	Hard-to-heal venous leg ulcers	Polyvinyl alcohol-based solution for administration in the wound bed	
PXL01	Derived from lactoferricin (human)	Phase II	Prevention of post-surgical adhesion formation in hand surgery	Hyaluronic acid-based hydrogel for administration at the surgical site	NCT01022242
Iseganan (IB-367)	Derived from protegrin 1 (porcine leukocytes)	Phase III	Oral mucositis in patients receiving radiotherapy for head and neck malignancy	Oral solution	NCT00022373
PAC-113	Derived from histatin 3 (human saliva)	Phase II	Oral candidiasis in HIV seropositive patients	Mouthrinse	NCT00659971

*α-MSH, α-melanocyte-stimulating hormone*.

Notably, several AMPs are currently under clinical development for therapeutic indications other than antimicrobials or antifungal agents. One of the most well-known of these peptides is LL-37, which has recently been evaluated in a phase I/II clinical trial as a local treatment to enhance healing of venous leg ulcers (Grönberg et al., 2014). The mechanisms by which LL-37 promotes wound healing are not fully understood, but are likely to involve several wound repair components such as re-epithelialization, angiogenesis, and inflammation. Reepithelialization is likely stimulated via chemoattractant effects of LL-37 on epithelial cells (Shaykhiev et al., 2005; Tokumaru et al., 2005) while vascularization is thought to be regulated by LL-37 stimulating endothelial tube formation and production of angiogenic factors (Lee et al., 2008; Rodríguez-Martinez et al., 2008). LL-37 exerts also chemoattractant effect on inflammatory cells (Chertov et al., 1996) and regulates secretion of proinflammatory cytokines (Scott et al., 2002; Niyonsaba et al., 2005; Mookherjee et al., 2006). Another AMP currently in clinical development for its properties other than anti-infection is PXL01. PXL01, which similarly to hLF1-11 is derived from human lactoferricin, has been evaluated in a hyaluronic acid-based gel formulation in a phase II clinical trial for prevention of post-surgical adhesion formation in connection to hand surgery (NCT01022242) (Wiig et al., 2014). PXL01 exhibits an inhibitory effect on the most important hallmarks of adhesion formation by repressing secretion of proinflammatory cytokines and promoting fibrinolysis (Nilsson et al., 2009). In addition, recent studies have shown that PXL01 regulates the production of the mucinous glycoprotein lubricin (Taguchi et al., 2009; Hayashi et al., 2013) in connection to surgery, which provides an additional mechanism to contribute to its adhesion-preventive properties (Edsfeldt et al., 2016).

Aside from direct administration of AMPs, there are several attempts ongoing to use agents to increase the endogenous production of AMPs by the body in order to boost the innate immune responses and thereby combat infections. As one example, vitamin D3 has been shown to directly modulate expression of several AMPs (Wang et al., 2004; Weber et al., 2005) and vitamin D supplements are now evaluated for their applicability for the treatment for bacterial infections in several ongoing trials (Yamshchikov et al., 2009).

## Challenges to develop AMPs for clinical applications

In spite of large number of AMPs going through clinical development, there is still a considerable discrepancy between the list of AMPs claimed as potent drug candidates in the patents or related scientific articles and the real outcomes of the clinical trials (Kosikowska and Lesner, 2016). Below, some of the technical, regulatory, and commercial challenges to bring AMP-based drugs into the clinical development are highlighted.

The design and optimization of therapeutic AMPs for treatment of infections usually starts with *in vitro* screening of the constellation of known or predicted peptide sequences for their antibacterial and/or antifungal properties using standard minimal inhibitory concentration (MIC) or minimal microbicidal concentration (MMC) assays (Fjell et al., 2012). However, the antimicrobial and antifungal activity of the AMPs is highly sensitive to environmental conditions, which results in discrepancies between *in vitro* vs. *in vivo* efficacy and makes the accurate prediction of anti-infection properties in clinical situation very difficult. Numerous reports describe AMPs with the desired *in vivo* antimicrobial effect demonstrated in relevant experimental animal models, while peptides appear to be inactive, or minimally active, when their efficacy was evaluated in MIC/MMC assays in the presence of physiologic salt concentrations and/or serum (Goldman et al., 1997; Bals et al., 1998; Ciornei et al., 2005; Dorschner et al., 2006; Chennupati et al., 2009; Björn et al., 2012; Myhrman et al., 2013; Rivas-Santiago et al., 2013; Maiti et al., 2014). Moreover, a number of naturally occurring AMPs are present in their native environment at concentrations, which do not kill bacteria *in vitro* (Dorschner et al., 2001). Several possible explanations for this apparent paradox have been put forward. It has been suggested that bacterial susceptibility to AMPs may be significantly higher in the mammalian ionic environment, which is not replicated in MIC/MMC assays. For example, when grown in the presence of carbonate, a ubiquitous molecule in many microenvironments of the body, both Gram-positive and Gram-negative bacteria show dramatically increased AMP sensitivity (Dorschner et al., 2006). It has also been proposed that the *in vivo* antibacterial activity of some AMPs is mediated primarily through their immunomodulatory effects rather than direct bacterial killing (Hancock and Sahl, 2006). On the other hand, AMPs displaying low MIC/MMC values *in vitro* may lack activity *in vivo* due to their rapid proteolytic degradation and/or protein binding in the body. In summary, the poor correlation between *in vitro* antimicrobial activity of AMPs and their *in vivo* efficacy is one of the technical obstacles, which has hampered the progression of these drug candidates toward clinical development.

Low metabolic stability of AMPs, which is an inherent risk of therapeutic peptides in general, is considered another key factor limiting their clinical application. Peptide drugs are generally characterized by low oral bioavailability due to pre-systemic enzymatic degradation and poor penetration of the intestinal mucosa, which makes their oral administration usually not possible (Vlieghe et al., 2010). Furthermore, systemic administration of peptides by, e.g., intravenous injection, is limited by a short half-life because of rapid degradation by proteolytic enzymes in blood plasma and rapid removal from the circulation by the liver (hepatic clearance) and kidneys (renal clearance) (Vlieghe et al., 2010). Consequently, local application of AMPs is the most common administration route including delivery in dermal creams and emollients, administration at the wound bed or the site of the surgery, mucosal application as nasal spray and similar. However, even upon local delivery, peptides are prone to degradation by tissue proteolytic enzymes. To reduce the liability of degradation by peptidases/proteases, cyclization of the AMPs, incorporation of D-amino acids and non-natural amino-acid analogs, and peptide mimetics with different backbone structures, are widely used (Fjell et al., 2012). End-tagging by hydrophobic oligo amino acid stretches has also been shown to diminish sensitivity of AMPs for proteolytic degradation (Malmsten et al., 2011). Furthermore, blocking N- or C-terminal ends of the AMPs by modifications such as N-acetylation, N-pyroglutamate, or C-amidation is frequently used to increase resistance toward peptidases (Brinckerhoff et al., 1999; Rink et al., 2010).

The relatively high costs of goods sold (COGS) of AMPs, which applies to peptide therapeutics in general, is considered another limitation to hamper AMPs' competitiveness compared with small molecule drugs. It is generally estimated that the production cost of a 5000 Da molecular mass peptide exceeds the production cost of a 500 Da molecular mass small molecule by more than 10-fold (Bray, 2003). Solid phase peptide synthesis (SPPS), the most commonly used method for chemical synthesis of therapeutic peptides (Amblard et al., 2006), is generally considered as the most mature technology available, at least for production of peptides with up to 50 amino acid residues (Raibaut et al., 2015). Production systems for recombinant peptides including bacteria, yeast, insect, and mammalian cells are alternatives to chemical synthesis; however, they typically require a long and expensive R&D phase and have limitations in relation to ability to introduce modifications into the peptide sequence. Although most of the AMPs, being relatively short, are produced by chemical synthesis, several AMPs such as a fungal defensin plectasin variant AP114 (formerly NZ2114), once developed by Novozymes for treating Gram-positive bacterial infections, and now owned by Adenium Biotech, are produced using a recombinant route (Mygind et al., 2005).

Regulatory hurdles may also considerably delay the clinical development of AMPs. Notably, some of the most well characterized AMPs—omiganan and pexiganan—have during their clinical testing run into regulatory barriers (Fox, 2013). Importantly, recognizing the decline in the approval of new anti-infectious agents, in combination with the alarming rise in resistance toward conventional antibiotics, has resulted in recent initiatives at governmental level as well as by regulatory authorities to facilitate development of novel anti-infectives such as additional years of market exclusivity and more flexibility with respect to clinical trial design (Fox, 2013). Interestingly, from a regulatory perspective, the peptide therapeutics tend to be considered as being a “mix” of both classical small molecules and biologics, since they are synthetic molecules but based on or having the same mechanism of action as endogenous proteins/peptides. The balance in this “mix” may differ in different territories, which makes the regulatory landscape, especially in relation to chemistry, manufacturing, and controls (CMC), more complex.

In relation to the safety profile of AMPs, only very few publications describing standardized toxicology data sets for AMPs are available (Björn et al., 2015). Moreover, given the low number of AMPs in clinical practice, the characterization of adverse effects in connection to administration in humans is limited with the exception of polymyxins, where systematic meta-analysis of safety is available and high incidence of nephrotoxicity and neurotoxicity associated with intravenous administration has been reported (Falagas and Kasiakou, 2006). In general, peptide therapeutics are considered to have advantages from the safety perspective compared to small molecule drugs since their degradation products are natural amino acids and, because of their short half-life, few peptides accumulate in tissues. Altogether, this reduces the safety risk and risk of complications caused by metabolites (Vlieghe et al., 2010). Notably, therapeutic peptides, even synthetic ones, are generally less immunogenic than recombinant proteins and antibodies (McGregor, 2008). Finally, local administration, which is the most common delivery route for AMPs, further reduces the risk for any systemic toxicology concerns.

## Innovative formulation strategies for AMPs

The stability, safety, and efficacy of AMPs can be further improved through innovative formulation strategies and design of drug delivery systems. Until now, only limited attention has been paid to this area although formulation offers the possibility to target the delivery of AMPs to a specific site with controlled release over time, thus minimizing side-effects and increasing efficacy (Eckert, 2011). Particularly interesting in this context is the use of nanocarriers, which have unique advantages due to their large surface area for adsorption/encapsulation of AMPs and prevention of self-aggregation of the peptides. Furthermore, nanostructured materials enable the design of formulations for local delivery to specific tissues and, by controlling degradation of the carrier, allow time-controlled release of the peptides, in addition to improving metabolic as well as chemical stability of the AMPs (Zhang et al., 2010; Witting et al., 2015; Sandreschi et al., 2016). Importantly, nanocarriers can be prepared from biocompatible and biodegradable materials such as lipids (e.g., phospholipids, triglycerides, cholesterol, and monoolein) and polymers [e.g., cellulose, chitosan, hyaluronic acid, poly lactic-co-glycolic acid (PLGA), and poly lactic acid (PLA)].

Several types of nanocarriers have been evaluated for delivery of AMPs with promising results. Hyaluronic acid nanogels were recently shown to successfully encapsulate the LL-37 analog LLKKK18 and enhance the killing of mycobacteria compared with the peptide alone both *in vitro* and *in vivo* (Silva et al., 2016), demonstrating a potential for treatment of tuberculosis. Importantly, the hyaluronic acid nanogels were also able to stabilize the peptide against proteolytic degradation and reduce the toxicity against host cells (Silva et al., 2016). PLGA nanoparticles have also shown potential in pulmonary delivery of cationic AMPs for the treatment of cystic fibrosis, where transport through the mucus layer and eradication of *Pseudomonas aeruginosa* biofilm *in vitro* could be enhanced by surface modification of the particles (d'Angelo et al., 2015). Mesoporous silica nanoparticles (MSN) exhibit several advantageous characteristics for encapsulation of AMPs due to their well-defined large surface area pores and the possibility to modify the surface properties, allowing fine-tuning of the release pattern (Vallet-Regí et al., 2007). It has been reported that the MSN surface properties and porosity influence the distribution of LL-37 in/at the surface of the particles, which in turn has a pronounced effect on the membrane adsorption, antimicrobial effect, and toxicity against eukaryotic cells of LL-37 as well as the ability of the particles to protect the peptide from proteolytic degradation (Braun et al., 2016). Furthermore, controlled-release of LL-37 from MSNs has been demonstrated through incorporation into mesoporous silica membranes that offers opportunities as surface coatings for implants (Izquierdo-Barba et al., 2009). Recently, liquid crystalline lipid nanoparticles and lipid nanocapsules have been investigated for encapsulation of several cationic AMPs varying in biophysical properties, with high loading efficacy and sustained or improved antimicrobial effect observed for many of these systems, suggesting potential for drug delivery (Boge et al., 2016; Umerska et al., 2016b). Notably, in addition to stabilizing the AMP and targeting delivery and release, nanocarrier material can have antimicrobial function on its own, thereby boosting the effect of the AMP (Malmsten, 2011; Umerska et al., 2016a).

To this date, nanoformulations as delivery systems for AMPs have only been evaluated *in vitro* and in experimental animal models, but intense development is now ongoing in relation to up-scaling and quality assurance of nanocarriers to bring these products into clinical phases.

## Conclusions

Overall, AMPs offer promising alternatives to standard therapies as anti-infectives and immunomodulatory agents with mechanisms of action which are less prone to resistance induction compared to conventional antibiotics. Although challenges in translating nonclinical candidate AMPs into successful clinical products are well recognized, the discovery and commercial development of next-generation therapeutic peptides and peptide mimetics is predicted to be accelerated by recent advances in overall understanding of their mechanism of action, resistance patterns, and smart formulation strategies. With several AMPs currently undergoing late stage clinical development in different therapeutic areas, the next years hold a promise to confirm the therapeutic benefit of these novel candidates and lead to market authorization of several new AMP-based drugs.

## Author contributions

All authors listed, have made substantial, direct and intellectual contribution to the work, and approved it for publication.

## Funding

This work was financially supported by the European Union's Seventh Framework Programme (FP7/2007-2013) under Grant Agreement No. 604182 within the FORMAMP project and by Promore Pharma AB, Solna, Sweden.

### Conflict of interest statement

MM is employee of Promore Pharma AB. The remaining authors declare that the research was conducted in the absence of any commercial or financial relationships that could be construed as a potential conflict of interest.
